# Implementation of a text messaging intervention to patients on warfarin therapy in Brazilian primary care units: a quasi-experimental study

**DOI:** 10.1186/s12875-022-01647-5

**Published:** 2022-03-23

**Authors:** Maira Viana Rego Souza-Silva, Mara Luiza de Paiva Domingues, Victor Schulthais Chagas, Daniella Nunes Pereira, Laura Caetano de Sá, Mychelle Stefany Santos Almeida, Thaís Lorenna Souza Sales, Magda César Raposo, Nathalia Sernizon Guimarães, João Antônio de Queiroz Oliveira, Antonio Luiz Pinho Ribeiro, Clareci Silva Cardoso, Maria Auxiliadora Parreiras Martins, Thais Bueno Enes, Thiago Barbabela de Castro Soares, André Oliveira Baldoni, Milena Soriano Marcolino

**Affiliations:** 1 The Post-graduate Program in Health Sciences: Infectious Diseases and Tropical Medicine, Medical School, Universidade Federal de Minas Gerais, Avenida Professor Alfredo Balena, 190, Belo Horizonte, 30130-100 Brazil; 2 Department of Internal Medicine, Medical School, Universidade Federal de Minas Gerais, Avenida Alfredo Balena, 190, room 246, Minas Gerais 30130-100 Belo Horizonte, Brasil; 3 The Graduate Program in Pharmaceutical Sciences, Universidade Federal de São João Del-Rei, R. Sebastião Gonçalves Coelho, 400, room 301, Divinópolis, 35501-296 Brazil; 4 Department of Medicine and Nursing – DEM, Universidade Federal de Viçosa, Av. Peter Henry Rolfs, University Campus, Viçosa, 36570-900 Brazil; 5 Medical School, Universidade Federal de Minas Gerais, Avenida Professor Alfredo Balena, 190, Belo Horizonte, 30130-100 Brazil; 6 Faculdade Ciências Médicas de Minas Gerais – FCMMG, Alameda Ezequiel Dias, 275, Belo Horizonte, 30130-110 Brazil; 7 The Graduate Program in Health Sciences, Universidade Federal de São João Del-Rei, R. Sebastião Gonçalves Coelho, 400, Divinópolis, 35501-296 Brazil; 8 Institute for Health Technology Assessment (IATS/ CNPq), Rua Ramiro Barcelos, 2359. Prédio 21 | Sala 507, Porto Alegre, 90035-903 Brazil; 9 University Hospital, Universidade Federal de Minas Gerais, Avenida Professor Alfredo Balena, 110, Belo Horizonte, 30130-110 Brazil; 10 Telehealth Center, University Hospital, Universidade Federal de Minas Gerais, Avenida Professor Alfredo Balena 110, room 107, Belo Horizonte, 30130-100 Brazil; 11 Universidade Federal de São João Del-Rei, R. Sebastião Gonçalves Coelho, 400, CEP 35501-296, Divinópolis, Brazil; 12 Department of Pharmaceutical Sciences, Universidade Federal de Minas Gerais, Avenida Antonio Carlos, 6627, Pampulha, Belo Horizonte, MG CEP 31270-901 Brazil; 13 Universidade Federal de Minas Gerais, Av. Professor Alfredo Balena, 110, CEP: 30130-100 Belo Horizonte, Brazil; 14 Universidade Federal de São João Del-Rei, R. Sebastião Gonçalves Coelho, 400, Divinópolis, CEP 35501-296 Brazil

**Keywords:** Warfarin, Anticoagulant, Cardiovascular disease, M-health, E-health, SMS, Text-messages, Digital health

## Abstract

**Background:**

Warfarin remains the most affordable oral anticoagulant in many countries. However, it may have serious side effects, and the success of the therapy depends on the patient’s understanding of the medication and their adherence to treatment. The use of short messages services (SMS) is a strategy that can be used to educate patients, but there are no studies evaluating this intervention in patients taking warfarin. Therefore, we aimed to develop, implement, and assess the feasibility of an intervention using SMS to primary care patients taking warfarin in a medium-sized Brazilian city.

**Methods:**

A bank of 79 SMS was drafted and validated by an expert panel. During 6 months, three times a week, patients received messages about anticoagulation with warfarin. At baseline and after 3 months, we assessed their knowledge and adherence with validated instruments. At the end of the follow-up, participants answered a satisfaction questionnaire. Subsequently, a scale-up phase was conducted, with another round of the intervention including 82 participants (29 from the first phase and 53 newly recruited). Seven months after the end of the scale-up, we asked the patients for their insights about the long-term effects of this program. All patients signed informed consent. The study was approved by the Research and Ethics committee of the *Universidade Federal de Minas Gerais*.

**Results:**

In the pilot, 33 (89.2%) patients completed the follow-up. Among the participants who answered the satisfaction questionnaire (*n* = 29), 86.2% considered that the intervention motivated a healthy lifestyle and improved their understanding of warfarin therapy. All patients were willing to continue receiving the messages. Adherence measured by the Measure of Adherence to Treatment (MAT) test was high in the pre-intervention assessment and remained high (96.7% vs. 93.3%; *p* = 1.0000). The proportion of patients who achieved > 75% correct answers on the Oral Anticoagulation Knowledge (OAK) test increased from 6.5% to 25.6, *p* = 0.0703. In the scale-up, 23 patients answered the long-term assessment questionnaire. The main long-term knowledge reported was dietary information. Nine patients received the messages but did not remember their content.

**Conclusion:**

The intervention was well-accepted and had a positive impact on patient’s knowledge about oral anticoagulation therapy. The scale-up assessment reinforced the need to constantly monitor digital interventions.

**Supplementary Information:**

The online version contains supplementary material available at 10.1186/s12875-022-01647-5.

## Background

Cardiovascular diseases (CVD) are the main cause of morbidity and mortality worldwide, with 17.5 million deaths reported in 2019 [1]. Of these, 75% occurred in low- and middle-income countries (LMICs) [1]. Acute myocardial infarction (AMI), stroke and venous thromboembolism (VTE) stand out among the major causes of CVD [1, 2].

Oral anticoagulant therapy is essential for the management of most cases of VTE and cardioembolic stroke to prevent additional thromboembolic events. Despite the development of the direct oral anticoagulants (DOACs), warfarin is still the most affordable oral anticoagulant in many countries [3]. For instance, in Brazil, DOACs are only available for out-of-pocket treatment while warfarin is fully funded by the public healthcare system (Sistema Único de Saúde – SUS). In addition, it is the first-line option for patients with some cardiovascular conditions, such as mechanical valve replacement [4].

Since warfarin’s therapeutic range is narrow and it may interact with different types of food and drugs, it has a risk of serious adverse events [5]. Thus, patients should be closely monitored with the prothrombin time (PT), expressed by the International Normalized Ratio (INR) test [5].

In this context, patient education is an essential strategy for the success of warfarin therapy [6]. Previous studies found an association of patients’ education with increased time within the therapeutic INR target (TTR) and lower incidence of complications [6–8]. However, patient’s adherence and understanding of warfarin therapy is often a challenge in clinical practice [9]. Hence, it is essential to seek novel strategies to enhance the odds of success with this treatment.

For this purpose, text messaging via short message service (SMS) is one of the strategies used for health promotion and education in different scenarios. Previous research showed this type of intervention could increase adherence to medications [10], attendance to medical appointments [11], modification of cardiovascular risk factors [12], weight loss in obese and overweight patients [13, 14], smoking cessation [15], and diabetes control [16].

Although the use of text-messaging as a health education strategy has increased, no studies evaluated this intervention on patients taking warfarin. In addition, little information is available about the use of this strategy in LMICs, such as Brazil, where the low level of education, health and digital literacy may make it less feasible to implement digital health interventions [17]. Therefore, we aimed to develop, implement, and assess the feasibility of an intervention using SMS to primary care patients taking warfarin in a medium-sized Brazilian city.

## Methods

**Fig. 1:**
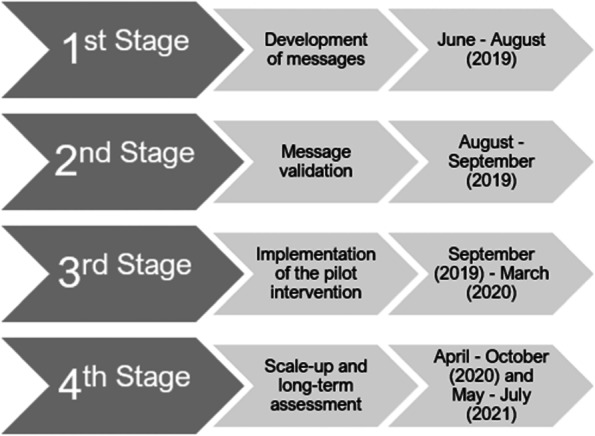
Flowchart of the methods employed in this study

This is a quasi-experimental study, which was executed in four phases: development, validation, pilot intervention, and scale-up (Fig. 1). This study was approved by the Research and Ethics committee of the *Universidade Federal de Minas Gerais (CAAE 86822518.0.0000.5149)*. All participants signed an informed consent form.

### First stage: development of the messages

In this phase, four healthcare professionals with clinical or academic experience in anticoagulation developed 79 messages. The content of all SMS was based on the guidelines from the American College of Chest Physicians [18], the American College of Cardiology/American Heart Association [19], the European Society of Cardiology [20], and the Brazilian Society of Cardiology [21]. Each message had a maximum of 145 characters. The content was divided into three main subjects: (1) diet, physical activity, and lifestyle, (2) adherence and motivation, and (3) general information about warfarin (which included basic concepts about the medication, drug interactions, follow-up control, and the identification of warning signs). All messages were semi-personalized, with a greeting customized with the patient’s name at the beginning of each text (Supplemental Box 1).

### Second stage: validation of the messages

**Fig. 2:**
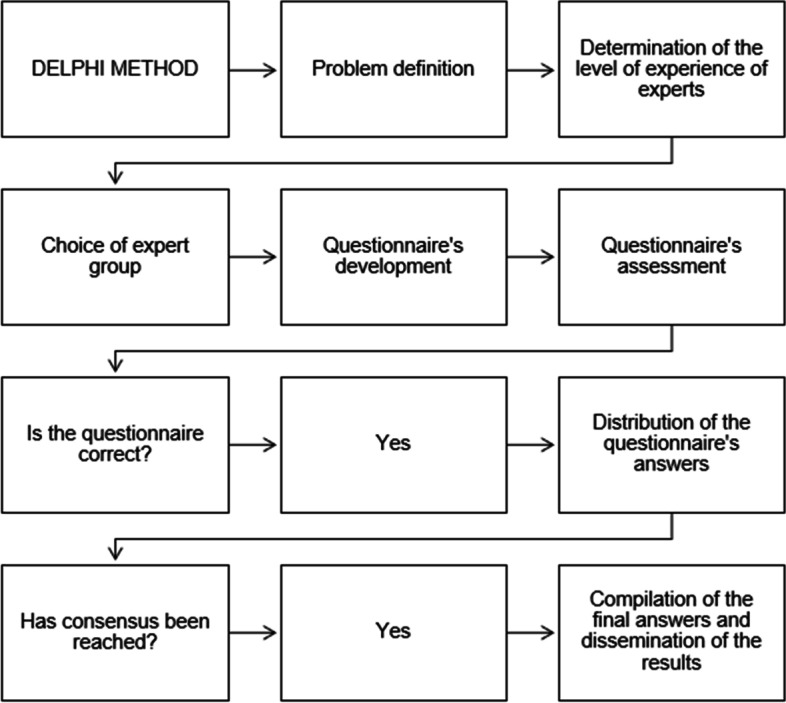
Flowchart of the steps of the Delphi methodology used to validate the content of SMS

In this stage, ten independent healthcare professionals with expertise on oral anticoagulation therapy and ten patients who took warfarin evaluated the messages using the Delphi method [22–24] (Fig. 2). This method has been used to achieve consensus in previous text-messaging studies [25, 26]. Healthcare professionals who were chosen were well-known experts on the topic at the city where the intervention took place, as well as experts from the anticoagulation clinic at the University Hospital, Universidade Federal de Minas Gerais, and this university was the coordination center of the research project. Two patients with long-term indication for anticoagulation and who had been taking warfarin for over a year were recruited from the local primary care unit in the city of Divinopolis (intervention site), the other eight were from the aforementioned anticoagulation clinic.

The Delphi technique was used to validate the SMS that were prepared by the authors, based on the available literature [22–26]. A Likert-scale questionnaire was elaborated with answers ranging from zero to ten, in which zero represented “completely disagree” and ten “completely agree”. There was also an additional open question for suggestions. The questions were about the suitability and usability of each section of the text-messages (Supplemental Table 1). Each evaluator had access to the bank with the messages and the questionnaire and in a final section they could make considerations about each message individually.

### Third stage: pilot intervention

The pilot intervention site was the city of Divinopolis, a medium-sized municipality in the state of Minas Gerais, Brazil. This city has 240,408 inhabitants (88% in the urban area of the city) and a human development index (IDH) of 0.764. Most of the inhabitants depend exclusively on the Brazilian public healthcare system. Thirty-two primary care units cover roughly 76% of the territory [27]. We invited the 15 units which had more than one patient taking warfarin to take part in the pilot.They were invited to participate by the field research team.

The pilot involved sending the messages to patients using an automated software developed in-house [14], capable of forwarding SMS simultaneously to multiple phone numbers at a scheduled calendar. Patients received messages free of all charges.

Patients on warfarin therapy who had an active registry within the primary care unit in the city of Divinopolis and a mobile phone capable of receiving text messages were included. Those who were dependent on daily living activities or were illiterate could be included if they had a caregiver who could read the text messages.

At baseline, trained researchers collected patients demographic and clinical data directly from the patients in the initial assessment or through the patient records within the primary care units. They also administered three instruments validated for the Brazilian population for the in-person pre-intervention assessment: 1) the Oral Anticoagulation Knowledge (OAK) Test [28, 29] to assess the level of patients’ knowledge on oral anticoagulation therapy; 2) the adaptation of the Measure of Adherence to Treatment to Oral Anticoagulation (MAT) [30] to assess the behavior and adherence of patients in the daily treatment; 3) and the Short Assessment of Health Literacy For Portuguese-Speaking Adults (SAHLPA-18) to assess their level of health literacy [31].

The OAK is a test that has a scale from one to 20 points, with each question scoring one point [28]. Previous studies suggested the classification of a patient’s level of knowledge according to the proportion of correct answers, as follows: < 50% as a low, 50 to 75% as an average, and > 75% as a high level of knowledge about oral anticoagulation [32]. The MAT is a seven-question instrument with an ordinal scale from one to six points. In this test, the values obtained from the patients’ responses are added up and divided by the total number of items. A score below five means the patient is “non-adherent”, whereas scores of five or higher means the patient is “adherent” [30] . Finally, the SALPHA-18 contains a list of 18 items. Whilst patients with a score of less than 14 are classified with “inadequate literacy”, those with higher scores are classified with “adequate literacy” [31].

The intervention started with a welcome text message. Subsequently, a researcher called patients to guarantee they had received the initial message. After that, patients received the SMS three times a week for 6 months, at 5 pm. We chose this time because this is when Brazilian doctors usually advise patients to take warfarin. They received one message a week from each category (Supplemental Box 1).

Researchers monitored the intervention by reviewing the reports generated by the automatic software and calling patients to ensure they were receiving the messages, as recommended [33]. We used the communication platform Zenvia®, which allowed us to generate reports. Once we identified any errors, we tried to call patients or contact the primary care unit where the patient was registered to check if they had changed the mobile number or had another problem to report.

Three months later, the OAK and MAT tests were redone. As the implementation of the text-messaging intervention was part of a larger project using other telehealth approaches, we had scheduled other interventions in the fourth month after the beginning of the SMS project. To avoid confounders, we re-applied the OAK and MAT before the beginning of other steps*.* After the end of the intervention, patients were contacted by telephone to answer a multiple-choice questionnaire with 14 questions to assess acceptability, usability, satisfaction, and self-perception of the impact of the SMS on their daily life. One open-ended question evaluated what dietary changes they had made (for those who answered that they had done dietary changes). At the end, there was an open space for suggestions. A word cloud was developed using an online software available in the website “Word Cloud Generator (by *MonkeyLearn*Ⓡ)” to show the most cited terms about dietary changes.

As part of the study protocol, focus groups would have been the final stage of this phase. However, as this approach required face-to-face interaction between researchers and participants and patients had limited access to videoconferencing, it was suspended due to the COVID-19 pandemic.

### Fourth stage: scale-up

In this phase, patients who reported in the satisfaction questionnaire they wanted to continue receiving text messages (*n* = 29) along with other patients who were recruited to the project after the beginning of the first round (*n* = 53) were included, as a scale-up phase. We included the same messages developed for the first round, and new messages developed by the same research team regarding COVID-19 (Supplementary Box 3).

This stage was implemented from April to October 2020, and the messages were sent six times a week during 6 months at 5 pm. To simulate a real-life intervention, in the scale-up we did not monitor the delivery of messages monthly after the initial subscription as we did in the first round.

In this phase, we aimed to assess whether the patients received the SMS even without intensive monitoring by the team. If they confirmed recipiency, we conducted a telephone questionnaire 7 months after the end of the intervention to measure how much of the information patients recalled (Supplementary Box 2).

### Statistical analysis

Statistical analysis was performed using Stata software, version 13.0 and R software, version 4.03. For the descriptive analysis, we described categorical variables as absolute and relative frequency. Continuous variables were described using measures of central tendency (mean and standard deviation or median and quartiles), according to the result of the test of Shapiro-Wilk. The data from the long-term assessment questionnaire was analysed categorizing patients’ responses (absolute number and percentage) and describing their answers.

To assess the validation of the messages, we converted the evaluators’ answers into an agreement proportion from zero to one. The content validation coefficient (CVC) was used to estimate the agreement proportion, according to Hernández-Nieto [34]. For each question, we calculated the CVC considering the average of the grades (Mx), the initial CVC (CVCi), the error (Pei), and the final CVC (CVCc) (Supplementary Table 2). A sufficient CVCc was ≥0.8, while values below 0.8 indicated the need for a second round of assessment.

For the evaluation of the pre- and post- intervention tests, total scores and percentages of OAK test, MAT scale, and SAHLPA-18 before the intervention and after 3 months were calculated. We used the exact McNemar test to assess the proportion of patients who achieved scores of ≥75% in the OAK test, which indicates a high level of knowledge of warfarin therapy. These tests were done before the intervention and after 3 months. Similarly, this statistical test was also used to verify the proportion of adherent patients according to the result of the MAT scale. Level of statistical significance was set at 5%.

## Results

### Validation of the messages

The messages were considered easy to understand, with a satisfactory agreement coefficient (CVCs ≥0.81). All parameters had a CVCc over 0.80, thus the second round of evaluation was not necessary. There was a strong agreement between professionals and patients’ perceptions (CVCt = 0.90). Some messages were adjusted according to evaluators’ suggestions (Supplementary Table 4).

### Pilot study

**Fig. 3:**
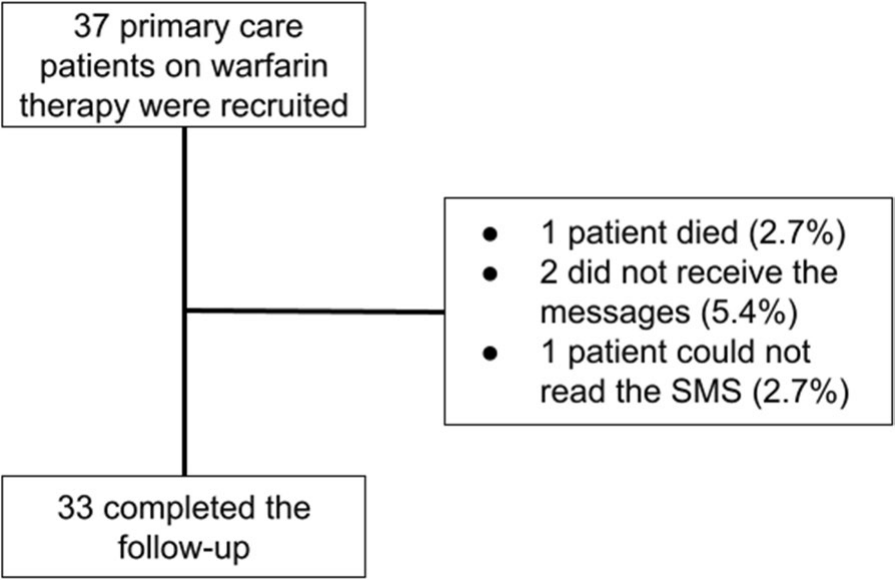
Flowchart summarizing the patients who completed the follow-up of the pilot study

From 37 patients who were included, 33 completed the follow-up (86.8%) (Fig. 3). From the patients who were excluded, one died, two did not receive the messages because they had changed their mobile numbers, and one assumed the ability of reading, but on the monthly monitoring reported difficulty in reading the messages.

Patients’ mean age was 60.0 ± 10.6 years-old and 18 (54.5%) were female. Most patients (72.6%) had a monthly personal income of less than four minimum wages, with 24.2% living with less than one minimum wage. Most patients had one to 8 years of formal education (57.8%), as shown in Table 1.

**Table 1:** Characteristics of the participants in the pilot study (*n* = 33)

Characteristics	
**Personal monthly income (Brazilian minimum wage**^**a**^**)**	**n (%)**
Less than one minimum wage	8 (24.2)
Between one and two minimum wages	8 (24.2)
Between two and four minimum wages	8 (24.2)
Between four and ten minimum wages	1 (2.7)
Refused to declare	8 (24.2)
**Level of education**	
1–8 years of formal study (elementary school)	19 (57.8)
9–11 years of formal study (secondary school)	5 (15.2)
Higher education	3 (9.1)
No formal study	2 (6.1)
Refused to declare	4 (12.1)
**Indication of anticoagulation**	
Venous thromboembolism	10 (30.3)
Atrial fibrillation	8 (24.2)
Mechanical heart valve replacement	9 (27.3)
Unspecified cardiovascular condition	6 (18.2)
**INR target range for warfarin therapy**	
2.0–3.0	24 (72.7)
2.5–3.5	9 (27.3)
**Risk of bleeding**^**b**^	
Low	10 (30.3)
Average	19 (57.6)
High	4 (12.1)
**Risk of thromboembolic event**^**c**^	
Low	20 (60.6)
Average	4 (12.1)
High	3 (9.1)
Very high	3 (9.1)
Information not available	3 (9.1)

*INR* international normalized ratio

^a^In 2020, Brazilian minimum wage was R$ 1.045,00, the equivalent to US$ 207,50 on 08 Jun 2021

^b^ Calculated using the HAS-BLED score: scoring system to assess the risk of major bleeding in patients taking anticoagulants. The scoring system is described in Supplementary Box 4. ^c^Calculated using the CHA2DS2-VASC score: clinical prediction system to estimate the risk of stroke in patients with non-rheumatic atrial fibrillation. The scoring system is described in Supplementary Box 5

The indication of oral anticoagulation therapy with warfarin was venous thromboembolism (30.3%), mechanical heart valve replacement (27.3%) or atrial fibrillation (24.2%). Around 18.0% of them were not aware of the indication of anticoagulation, nor the information could be retrieved by their medical records (Table 1).

At baseline and three-month follow-up, 30 participants completed the MAT scale, and 31 patients completed the OAK test. Adherence assessed by the MAT scale was high at baseline and remained high after 3 months (96.7% vs. 93.3%; *p* = 1.0000). With regards to the OAK test, the proportion of patients who achieved ≥75% of correct answers rose when comparing baseline and after 3 months (6.5% vs. 25.8%, *p* = 0.0703). The SAHLPA-18 test showed that most patients (60.6%) had inadequate health literacy (Table 2).

**Table 2:** Distribution of the answers of the three questionnaires before intervention and after 3 months the intervention had started in the pilot study

Questionnaire	Before intervention n (%)	After 3 months n (%)
**OAK test (*n*** **= 31)**^**a**^		
Low level of knowledge (> 50% of correct answers)	11 (35.5)	10 (32.3)
Average level of knowledge (50 to 75% of correct answers)	18 (58.1)	13 (41.9)
High level of knowledge (≥75% of correct answers)	2 (6.5)	8 (25.8)
**MAT scale (*n*** **= 30)**^**a**^		
Adherent	29 (96.7)	28 (93.3)
Non-adherent	1 (3.3)	2 (6.7)
**SAHLPA-18 test (*n*** **= 33)**		
Inadequate health literacy	20 (60.6)	–
Adequate health literacy	13 (39.4)	–

^a^Considering just patients who answered the questionnaire on both study times

*OAK* Oral Anticoagulation Knowledge, *MAT* Measure of Adherence to Treatment, *SAHLPA-18* Short Assessment of Health Literacy For Portuguese-Speaking Adults

### Patient’s satisfaction

Responses to the satisfaction questionnaire are shown in Table 3. Among the respondents, 89.7% found the messages very useful, 93.1% said they were easy to understand and 75.9% said they read all the messages. All respondents were willing to continue in the program and would recommend it to others.

**Table 3:** Responses of the satisfaction questionnaire (*n* = 29) in the pilot study

Questions and answers	n (%)
**Did you find the text messages you received on your mobile phone useful?**	
Very useful	26 (89.7)
Not very useful	2 (6.9)
Not useful	1 (3.4)
**Were most of the messages easy to understand?**	
Yes, easy	27 (93.1)
Neither easy or difficult	2 (6.9)
Difficult	0 (0.0)
**Did receiving these messages help you to change your lifestyle?**	
Helped a lot	18 (62.1)
Helped little	7 (24.1)
Did not help	4 (13.8)
**Out of every ten text messages you received, how many did you read?**	
Two to four messages	4 (13.8)
Five to eight messages	3 (10.3)
Nine or ten messages	22 (75.9)
**Did you follow the information of the text messages you received?**	
Yes, I always did	17 (58.6)
Yes, I followed many times, but not always	10 (34.5)
Yes, I did, but rarely	2 (6.9)
Never followed	0 (0.0)
**Have you understood better about treatment with Marevan after receiving the messages?**	
Yes, I understand a lot better	25 (86.2)
Yes, I understand a little better	2 (6.9)
No	2 (6.9)
**Did the messages help you remember to take Marevan?**	
Yes, Always	16 (55.2)
Yes, often, but not always	6 (20.7)
Yes, but rarely	3 (10.4)
No	4 (13.8)
**Did the messages cause you to change your diet?**	
Yes	22 (75.9)
**Did you immediately delete the text messages you received?**	
No	21 (72.4)
**Did you show or share the messages you received with a friend or a relative?**	
No	18 (62.1)
**The number of messages you received per week was:**	
High	1 (3.4)
Adequate	27 (93.1)
Low	1 (3.4)
**If this study continued, would you like to continue receiving messages?**	
Yes, daily	4 (13.8)
Yes, 3 to 5 times a week	19 (65.5)
Yes, 1 to 2 times a week	6 (20.7)
No	0 (0.0)
**Would you recommend this messaging system to a friend or a relative?**	
Yes	29 (100.0)

**Fig. 4:**
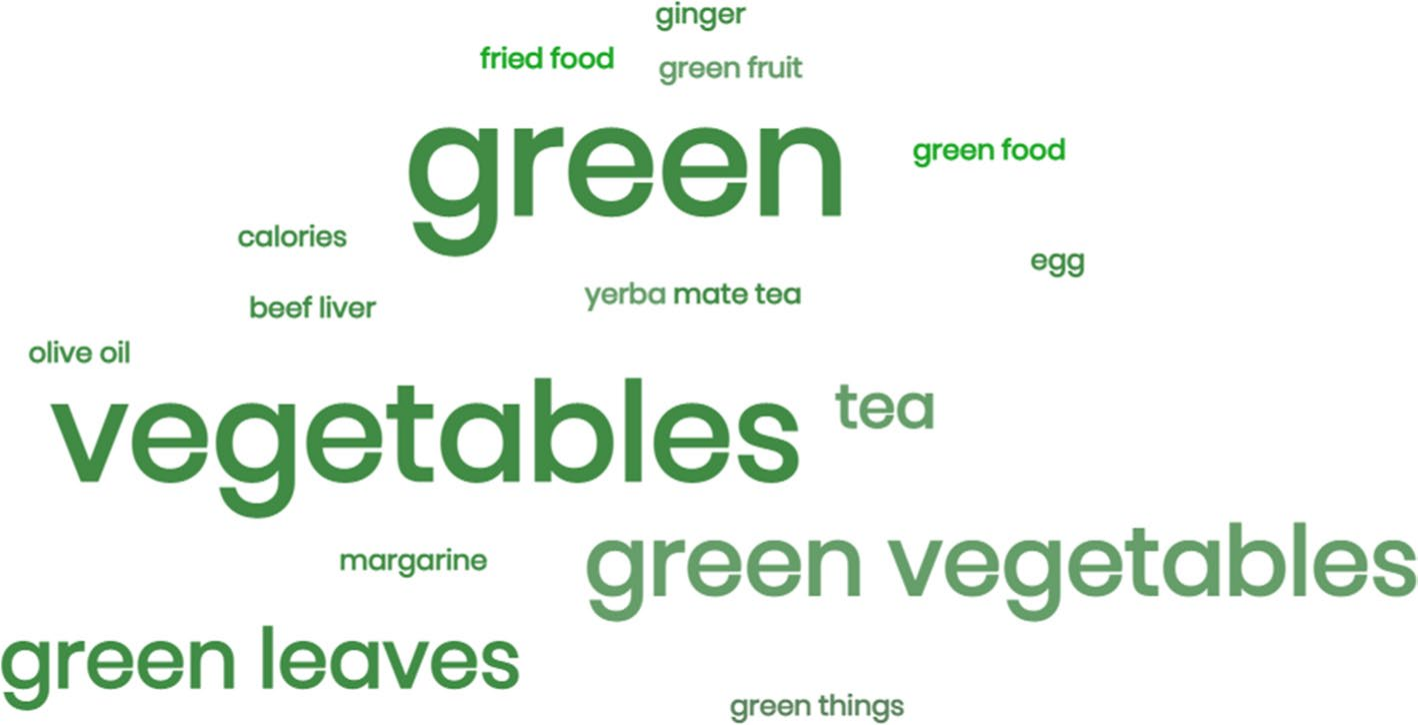
Word cloud with the most cited terms by patients when asked about dietary changes after the implementation of the program

As for their self-perception of behavioral changes, 86.2% said the messages helped to change their lifestyle and to better understand warfarin therapy. Approximately 76.2% reported changes in their diet. When asked about what dietary changes they had made, 16 patients (55.2%) answered changes in their consumption of green foods (leaves or vegetables) (Fig. 4).

### Scale-up and long-term assessment

**Fig. 5:**
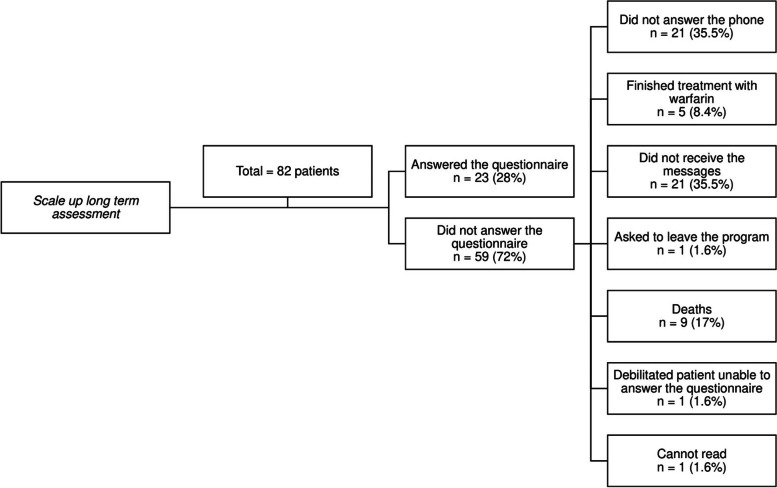
Flowchart showing patients who participated in the long-term scale-up intervention

The scale-up included 82 patients. Of those, 23 (28%) answered the long-term assessment questionnaire (Fig. 5).

The impact of the intervention on patients’ knowledge about dietary habits while taking warfarin was noted by the researchers, as:

Fourteen patients (60.9%) reported changes regarding their diet:

*What struck me the most was that I didn't know that olive oil could interfere with the warfarin. [...] It was surprising. I didn't know about the alcoholic beverages, but I don’t drink it.* [Male, 56. Indication for anticoagulation: mechanical heart valve prosthesis]

Twenty-two patients (95.7%) wanted to continue receiving messages. One patient also pointed out the possibility of sharing this information with other patients who take warfarin but were not enrolled in this program. This indicates a possibility of dissemination of the knowledge acquired from the intervention:

*It was good to receive the messages because I shared them with other people who were also taking warfarin but didn't have as much information about it.* [Female, 54. Indication of anticoagulation: mechanical heart valve prosthesis]

In addition, three patients (13.1%) highlighted they learned about the control of warfarin therapy using the INR test:

*Yes, I received many messages. I remember that olive oil and green vegetables may interfere with Marevan. Also, I now know that if my INR test is higher or lower than the limit, I should tell my doctor.* [Male, 56. Indication of anticoagulation: mechanical heart valve prosthesis]

However, one patient reported problems regarding reading or understanding the messages, and one patient cited visual impairment:

*[...] I am blind in my right eye and now I am waiting for surgery. This has made it difficult for me to read the messages. I would like to continue to receive the messages, but I can't read them. I would like to receive voice messages.* [Male. 54. Indication of anticoagulation: venous thromboembolic event]

Nine patients (39.1%) mentioned that they did not remember the content of the messages at the time of the long-term assessment:

*I received the messages, I remember they told me how to take the Marevan, but I don't remember any details.* [Male, 65. Indication of anticoagulation: chronic atrial fibrillation]

## Discussion

We described the development and implementation of a digital health intervention based on mobile text messaging to promote access to information about warfarin therapy. The text messages were validated with experts and patients and reached a CVC above 0.80 in the first round of assessment. We found that this intervention was well-accepted by primary care patients, most of them reported that it helped them to change their lifestyle and eating habits and affirmed that their knowledge on anticoagulation improved after receiving the SMS.

It is well-known that warfarin therapy has a narrow therapeutic index, wide variability, dose-response, and potential drug-drug and drug-food interactions. Therefore, the management of this treatment is challenging in the clinical practice. Periodic monitoring with INR tests is required to guide dose adjustments and reduce the risk of adverse events [5]. Educating patients regarding anticoagulation therapy is an essential step to success, to support their engagement to the treatment and improving self-care [7, 9]. In this intervention, the fact that 86% of the patients felt they had a greater knowledge of warfarin therapy following the messages, as well as the igreater proportion of patients who achieved ≥75% correct answers in the OAK test, demonstrate the impact of such strategies.

One important principle in this intervention was the elaboration of the text messages considering the translation of scientific evidence to a simpler and accessible language using only a limited number of characters. We chose the Delphi method to validate the messages which were scrutinized by patients on warfarin therapy and experts in the field [22–26]. This method allowed a multidisciplinary and collective construction of our bank of messages, which was very well-received by the evaluators and the CVC reached in the first round of evaluation did not require another round. In the early stages of the study development, we opted to include not only experts, but also the patients’ perception about the content of the messages. Patients’ views are fundamental in all stages of the process, as they are the target of this program. The early stages of text-messaging interventions involving the development of the content are often underreported.” [35]. However, as Ybarra et al. noted, behavior changes depend mostly on what is given (content) rather than how it is given (delivery format) [36]. Therefore, involving patients in the process to act as evaluators was valuable to better tailor it to the intended audience, as well as to understand how to communicate more effectively with them.

After the first round of the intervention (pilot), two patients lost access to their mobile phone numbers, and one gave up because he could not read the messages. In Brazil, it is common to change phone numbers, because of the affordability of prepaid phone plans. However, in the second phase, the proportion of patients who received the messages and completed the intervention was significantly lower. It is important to notice that the second phase took place during the first wave of the COVID-19 pandemic, and we had a higher proportion of patients who died or stopped using warfarin compared to the first phase. Periodic surveillance to ensure that patients are receiving text messages is recommended by the World Health Organization, and has shown in the present study to be essential for the success of this type of intervention [33].

After the pilot, in the post-intervention satisfaction evaluation, lifestyle changes were reported by around 86% of the patients. When analyzing these changes in-depth, almost 80% of patients reported changes in their food consumption habits. In the open-ended question inquiring them about these changes, most patients reported changes in the consumption of green foods, such as green leaves and other vegetables. Historically, healthcare professionals used to advise patients taking warfarin to completely remove green foods from their diet. This was a recommendation because these foods are sources of vitamin K, which can enhance the effect of warfarin, potentially leading to a higher incidence of hemorrhagic complications. However, the current body of evidence demonstrates that maintaining a consistent intake of vitamin K is more important than the quantity [37–40]. Even though this is the current recommendation, many patients and healthcare professionals are still unaware of this information. We believe that this is one of the reasons why most patients cited changes in their green foods’ consumption.

Regarding their overall knowledge about warfarin therapy, 86.2% of patients reported greater knowledge of their therapy in the satisfaction questionnaire. In the OAK test, there was an increase in the proportion of patients who achieved ≥75% of correct answers (6.5% vs. 25.8% *p* = 0.0703). Previous studies have shown that an improvement in knowledge is associated with increased time within the therapeutic range and lower incidence of complications [7, 9, 41]. A very high proportion of the patients were already adherent to the treatment, when assessed by the MAT test. Therefore, it was not possible to assess the impact on adherence.

On the questionnaire’s open question some patients reported difficulties reading the messages for two different reasons: (1) Reduced visual acuity - which affected their readability on the mobile phone screen; (2) Difficulties to comprehend the messages. To overcome these difficulties, they needed the help of others (such as caregivers or family members) who read the SMS content for them. In Brazil, although illiteracy has reduced over the last decades, it still is a social issue. A national survey conducted in 2016 showed that 11.8 million people could not read or write, and illiteracy increased with age and lower family income [42]. On the top of that, there are the individuals who are not illiterate, but still have low health literacy [43]. In our intervention, most participants were more than 60 years-old, lived with less than four minimum wages, and studied less than 8 years. This social context can explain why patients reported reading difficulties, and this is an important aspect to take it into consideration when planning further interventions for this target audience.

Interventions targeting people with inadequate health literacy should be designed to address functional abilities, such as numeracy and literacy skills. It should also incorporate a broader view of health literacy that comprises the resources and support a person needs to engage with and to develop desirable self-care behaviors to improve their health condition [44, 45]. To improve clinical outcomes among social vulnerable populations, health literacy interventions are more prone to succeed if they are theory-based, multifaceted and patient-centered taking into account the context of the individual (e.g. family), their sociocultural interactions and the healthcare [44, 45]. Regarding oral anticoagulation, established protocols for treatment management and tailored educational interventions have been reported to help overcome the barriers of inadequate health literacy [46, 47].

As part of the study protocol, focus groups would have been the final stage of the first phase of this project [48]. However, as this approach requires face-to-face interaction between researchers and participants, it was suspended due to the COVID-19 pandemic. Safety for both our researchers and participants (most of which were at-risk groups for severe COVID-19 disease) could not be guaranteed in this situation. Furthermore, the patients had limited access to videoconferencing tools that would enable us to adapt these interviews to a digital approach. Although this evaluation had to be postponed due to the pandemic, there is the expectation of resuming the groups to conduct a thorough evaluation of qualitative data.

The scale-up of the SMS project supplied the demands of the large number of patients who said they would like to keep receiving messages. We also included the ones who could not receive the SMS before because they were recruited after the pilot had started. This round was designed to test how the project would be in “real-life”, without constant monitoring by a research team. Due to the COVID-19 pandemic, we chose to incorporate new messages using the same know-how to give patients information about the novel disease. It shows that this type of intervention can be easily adjusted to incorporate new information using the same low-cost technology. In our experience, the lack of constant monitoring caused two pitfalls: (1) The automated system presented persistent failures to some of the patients, so they never received the messages; (2) Social problems were much more prevalent than they were in the first round, with reports of nine deaths and five patients who stopped to take warfarin. Although our intention was to test a real-life implementation, these issues could be easily identified if we had implemented a surveillance system as we did in the first round. This is an important lesson for the future, and is in accordance with the recommendation by the guideline of World Health Organization for digital interventions [33].

We also assessed the long-term impact the SMS had in the patients’ perception. Although it is known that the perceived positive effect of the intervention decreases with time, there is a lack of evidence analyzing the duration of these benefits [49, 50]. In our analysis, 9 (31%) of the patients could not recall any of the message’s content after 7 months of the end of the intervention. This assessment points out that, to be sustainable and scalable real-life interventions should have in mind the need for periodic rounds of messages, especially when targeting long-term chronic illness. Also in this phase, another interesting insight is that some patients referred to a feeling of “being taken care of”. This reflects that this type of intervention can have an emotional impact on patients that cannot be easily assessed by tests. Still in the qualitative analysis, one patient gave feedback that he would prefer the content to be presented to him by voice, because of his difficulty in reading. This suggestion is very relevant for the next steps of the project to make our intervention more accessible to patients who have limited literacy or visual impairments. Although voice-based interventions have not been extensively explored so far, some studies showed this strategy could improve health outcomes [51, 52]. Indeed, more studies testing this form of delivering information needs to be done.

This study of a digital intervention using SMS to primary care patients taking warfarin was very well accepted by patients and had an impact on their knowledge of warfarin therapy. To the best of our knowledge, this is the first study to describe a SMS intervention for this target audience. We also showed the lack of monitoring may compromise an intervention, and a surveillance strategy should be ensured even in scale-up projects. Finally, we believe that a strategy using voice-based messages would be an interesting way to overcome the barriers in the implementation of such strategies for elderly patients, who may have difficulty in reading small font-size SMS, and for those who have limited literacy, which is a very common situation in LMICs.

This study has limitations. In this intervention, our sample size was small, and further studies are needed to generalize our findings, as well as to assess the impact of the intervention on clinical outcomes. The satisfaction questionnaire is a self-perception, which can lead to bias. In addition, the variables we measured for assessing impact are not clinical, and we cannot claim that a better understanding of warfarin therapy according to the OAK test would lead to a better clinical management. As most participants were adherent to medication according to the MAT test before the intervention the impact of the strategy on adherence could not be assessed.

## Conclusion

This intervention was well-accepted and had a positive impact on patients’ knowledge about oral anticoagulation therapy. All patients who answered the final questionnaire showed interest in continuing to receive the messages. The scale-up phase presented important delivery pitfalls that highlighted the need to constantly monitor digital interventions. In the long-term assessment, we showed that the perceived benefit reduced with time and that periodical rounds could be necessary for a sustainable long-term program. Finally, we also learnt that different delivering approaches, such as using voice-based interventions, may be an interesting strategy overcome low digital and health literacy.

## Supplementary Information

**Additional file 1: Supplementary Table 1**. Evaluation Questionnaire According to the Likert Scale. **Supplementary Table 2**. Content validation coefficient formula description used to validate the text messages. **Supplementary Table 3**. Content validation coefficient of text messages validated by professionals and patients *. **Supplementary Table 4**. Suggestions and changes for the messages. **Supplementary Box 1**. Translation of the messages sent to warfarin users. **Supplementary Box 2**. Long-term interview. **Supplementary Box 3**. Examples of translated COVID-19 SMS sent to patients. **Supplementary Box 4**. Description of the HAS-BLED score. **Supplementary Box 5**. Description of the CHA2DS2-VASc score. **Supplementary Box 6**. Examples from guidelines to final messages.

## Data Availability

The datasets used and/or analyzed during the current study are available from the corresponding author on reasonable request.
