# Supplementary material for: Implementation of a text messaging intervention to patients on warfarin therapy in Brazilian primary care units: a quasi-experimental study
Source: BMC Prim Care. 2022 Mar 23;23:54. doi: 10.1186/s12875-022-01647-5 (PMC8942053; doi:10.1186/s12875-022-01647-5)
Supplement: Supplementary file 1 — Additional file 1: Supplementary Table 1. Evaluation Questionnaire According to the Likert Scale. Supplementary Table 2. Content validation coefficient formula description used to validate the text messages. Supplementary Table 3. Content validation coefficient of text messages validated by professionals and patients *. Supplementary Table 4. Suggestions and changes for the messages. Supplementary Box 1. Translation of the messages sent to warfarin users. Supplementary Box 2. Long-term interview. Supplementary Box 3. Examples of translated COVID-19 SMS sent to patients. Supplementary Box 4. Description of the HAS-BLED score. Supplementary Box 5. Description of the CHA2DS2-VASc score. Supplementary Box 6. Examples from guidelines to final messages. [file 12875_2022_1647_MOESM1_ESM.docx]

**Supplementary Materials**

**Supplementary Table 1.** Evaluation Questionnaire According to the Likert Scale

| **Evaluation of Text Messages** |
| --- |
| Do the messages follow a logical sequence? |
| Is the language of the messages adequate? |
| Are the messages concerning indications for Marevan® adequate? |
| Do the messages regarding the importance of the use of Marevan® help patient adhesion and correct use of the medication? |
| Do the messages regarding the pharmacology of Marevan® guide the patient about its action and help in adhesion? |
| Do messages regarding diet effects on Marevan® show the need of maintaining a satisfactory diet? |
| Do messages regarding Marevan®’s interaction with other medications clarify the importance of not self-medicating? |
| Do the messages concerning INR exams help the patients to understand the need of doing follow-up exams? |
| Do the INR exams messages help patients to understand the importance of taking recent INR results to their appointments? |
| Can messages contribute as a tool to a patient's self-care? |

**Supplementary Table 2.** Content validation coefficient formula description used to validate the text messages.

| **CVC’s Formula** | | | |
| --- | --- | --- | --- |
| 1) M_x_= ∑x/ J | 2) CVCi= Mx/ V_max_ | 3) CVCc= CVCi – (1/J)^J^ | 4) CVCt= Mcvci – M(1/J)^J^ |
| Average of each item: sum of values obtained by the Likert scale (x) by the number of judges (J) | Item’s CVC: average of the item by the maximum value that the item could achieve | CVCc: item’s CVC deducted from the constant (Pei) | Total CVC: item’s CVC average deducted from the constant average |

CVCi: content validation coefficient of each item; CVCc: content validation coefficient of each item deducted from the constant; Pei: formula’s constant; CVCt: criteria’s total content validation coefficient.

**Supplementary Table 3.** Content validation coefficient of text messages validated by professionals and patients *

| **Questions for Evaluation** | **CvCc**  **Professionals** | **CvCc**  **Patients** |
| --- | --- | --- |
| Do the messages follow a logical sequence? | 0.92 | 0.96 |
| Is the language of the messages adequate? | 0.83 | 0.99 |
| Are the messages regarding the indications for Marevan® use adequate? | 0.92 | 0.97 |
| Do the messages regarding the importance of the use of Marevan® help patient adhesion and correct use of the medication? | 0.94 | 0.97 |
| Do the messages regarding the pharmacology of Marevan® guide the patient about its action and help in adhesion? | 0.94 | 0.97 |
| Do messages regarding diet effects on Marevan® show the need of maintaining a satisfactory diet? | 0.87 | ii 0.99 |
| Do messages regarding interactions of Marevan® with other medications clarify the importance of not performing self-medication? | 0.86 | 0.97 |
| Do the messages concerning INR monitoring help the patients to understand the need of doing follow-up exams? | 0.90 | 0.97 |
| Do the INR monitoring messages help patients to understand the importance of taking recent INR results to their appointments? | 0.97 | 0.98 |
| Can messages contribute as a tool to a patient's empowerment? | 0.85 | 0.97 |

CvCc: content validation coefficient of each item deducted from the constant; INR: International Normalized Ratio

**Supplementary Table 4.** Suggestions and changes for the messages

| **Suggestions** | **Changed? (Yes/No)** |
| --- | --- |
| Substitute INR* for PT* | No |
| Use only one name for the medication, warfarin or Marevan® | Yes |
| Use the term hemorrhage instead of bleeding | No |
| Substitute the term “thin” to “make the blood thinner” | Yes |
| Give examples of anti-inflammatory medication | Yes |
| Add messages concerning why the patient should do the INR exams and its frequency | Yes |

Some of the suggestions were considered, but not changed, due to the lack of patients' knowledge of the suggested medical terms, such as using the Portuguese word for "hemorrhage" instead of "bleeding" and "PT" instead of "INR". The use of the name Marevan® instead of warfarin when referring to the medication was defined according to patients' knowledge, since most of them only knew the medication by the commercial name. *INR= international normalized ratio; *PT= prothrombin time

**Supplementary Box 1.** Translation of the messages sent to warfarin users

| **Presenting the messaging program:**  1) Hello (patient's name)! Starting today, you will receive messages with information about warfarin. Knowing the medicine helps in the success of the treatment!  2) Hello (patient's name)! Marevan® and Coumadin® are trade names. Warfarin, present in them, has an anticoagulant function. Both are the same medicine! |
| --- |
| **General Information/ Concepts/ Drug interactions /Alarm Signals:**  1) Hello (patient's name)! Do you know what Marevan® is used for? It is used to make the blood thinner to prevent clot formation.  2) Hello (patient's name)! Did you know that anticoagulation is the name of the treatment given to people who use Marevan® or other medicines to thin the blood?  3) Hello (patient's name)! Did you know that blood clots can clog veins? That is why it is important to take Marevan®, which prevents clots formation.  4) Hello (patient's name)! Some people who have heart arrhythmia will need to take Marevan® to prevent blood from getting thick and causing a brain stroke.  5) Hello (patient's name)! Marevan® is indicated for those who have had leg or lung embolism. Its use is to prevent such diseases from happening again.  6) Hello (patient's name)! Marevan® helps to prevent brain stroke, so it is important to always use the medicine correctly.  7) Hello (patient's name)! Did you know that Marevan® helps prevent diseases such as lung embolism and vein thrombosis? It is important to take this medicine correctly!  8) Hello (patient's name)! INR is a test to see how blood is clotting. It helps to adjust the dose of Marevan® to prevent bleeding and clot formation.  9) Hello (patient's name)! How often does the INR test need to be done? It depends on the case! Follow the guidance of the professional who controls it.  10) Hello (patient's name)! When the INR is within the recommended range (2.0 to 3.0) the blood is neither "thick" nor too "thin", this is ideal!*  11) Hello (patient's name)! When the INR is within the recommended range (2.5 to 3.5) the blood is neither "thick" nor too "thin", this is ideal!*  12) Hello (patient's name)! Did you know that when the INR is above the recommended range, blood that is too thin, which can cause bleeding?  13) Hello (patient's name)! Did you know that when the INR is below the recommended range there is a greater risk of blood clots?  14) Hello (patient's name)! Did you know the dose of Marevan® is individual and may vary from patient to patient, according to the result of the INR?  15) Hello (patient's name)! Always store Marevan® at room temperature (15 ° C to 30 ° C) and away from humidity. This helps to better preserve the medication!  16) Hello (patient's name)! Some people use Marevan® for life, while others only for a moment. Talk to your doctor about how your treatment will be!  17) Hello (patient's name)! Do you have any questions about your treatment with Marevan®? Ask the professional who controls it at your next appointment!  18) Hello (patient's name)! Always watch for signs of bleeding. If there is any, seek medical attention immediately!  19) Hello (patient's name)! Purple spots on the body, bleeding from the nose or mouth can be signs that the blood is too thin. Look for a doctor!  20) Hello (patient's name)! If you have blood in your urine, vomiting blood, black stools (like coffee grounds), seek emergency care immediately.  21) Hello (patient's name)! When collecting blood tests, always tell the person who is going to collect about the use of Marevan® to prevent increased bleeding.  22) Hello (patient's name)! Did you know that some drugs, such as anti-inflammatory drugs, if used with Marevan®, can increase the risk of bleeding?  23) Hello (patient's name)! Examples of anti-inflammatory remedies are ibuprofen, tenoxicam, diclofenac, nimesulide. Do not use medicine without professional guidance!  24) Hello (patient's name)! Taking medication on your own is not recommended, seek medical advice before using any medication that is not on the prescription.  25) Hello (patient's name)! Did you know that some antibiotics can interfere with the treatment with the anticoagulant? Never use medications without medical advice!  26) Hello (patient's name)! Did you know that the use of anti-inflammatory drugs can interfere with the treatment with anticoagulants? Never use medications without medical advice!  27) Hello (patient's name)! The medicine that is good for your neighbor or friend may not be right for you. Get information about it before using any medicine!  28) Hello (patient's name)! Will you be away from home at Marevan® time? Remember to take the medicine with you and take it on time! |
| **Adherence / motivation:**  1) Hello (patient's name)! We want to help you improve control of treatment with Marevan®! The messages can help you.  2) Hello (patient's name)! Have you remembered to take Marevan® today? It is important that you take your medicine every day. If you forget, never take the double dose.  3) Hello (patient's name)! If you ever forget to take your dose of Marevan®, remember to discuss it with your healthcare professional at your next appointment.  4) Hello (patient's name)! Remember to take Marevan® as directed by the professional who controls it and never change the dose on your own.  5) Hello (patient's name)! It is very important that you never stop taking Marevan® without first consulting your doctor, as it can cause health problems.  6) Hello (patient's name)! Always use Warfarin at the same time! We recommend the 17:00 (5:00 pm) time.  7) Hello (patient's name)! Success in treatment depends on you, take the same medication as in the prescription and have an adequate diet. You are capable!  8) Hello (patient's name)! It is important to take Marevan® according to the recipe! Taking it at the right time can help for better effect of the remedy.  9) Hello (patient's name)! Always follow the most recent guidance on the dose of Marevan®. The doses of the medicine can change between consultations.  10) Hello (patient's name)! If you need to have any surgery or tooth removal, notify your healthcare professional at least 1 week in advance.  11) Hello (patient's name)! When traveling, don't forget to take your medicine. Plan to do the INR out of your usual health unit.  12) Hello (patient's name)! Never fail to take the tests the doctor, pharmacist or nurse asks you for the return visit. Take care of your health.  13) Hello (patient's name)! Do not exchange your medications for others unknown or without medical advice.  14) Hello (patient's name)! We wish you a Merry Christmas! Never stop taking care of your health and know that we are always ready to help you!  15) Hello (patient's name)! Happy New Year! Start the year motivated to take better care of your health. Believe in yourself!  16) Hello (patient's name)! Always pay attention to the dosage of Marevan®. Some people may have to use different doses each day of the week.  17) Hello (patient's name)! Some people may have to take a broken pill, pay attention to that. Ask your healthcare team for guidance if you are unsure.  18) Hello (patient's name)! If you ever forget the dose of the medicine, do not use the double dose the next day. Take your daily pill at the usual time.  19) Hello (patient's name)! If you have questions about how to use Marevan®, ask for help! The health professional will always be able to guide you.  20) Hello (patient's name)! If you ever take too many Marevan® pills by accident, seek medical help even if you have not had any symptoms.  21) Hello (patient's name)! Always remember to renew your medication prescription before it runs out! Don't go even a day without using your medicine.  22) Hello (patient's name)! An interesting tip: set an alarm clock or alarm to remind you of the right time to take Marevan®!  23) Hello (patient's name)! Make a note of the date of return for Marevan® control and take a recent INR exam as instructed by the doctor.  24) Hello (patient's name)! Using warfarin can be tiring but be aware that it is a medicine that will help your health a lot. We are with you! |
| **Diet / physical exercise / life quality:**  1) Hello (patient's name)! Did you know that Marevan® can have an increased or decreased effect on your body due to the presence of vitamin K in some foods?  2) Hello (patient's name)! To help control the INR, eat foods that contain vitamin K regularly, such as green leafy vegetables.  3) Hello (patient's name)! And what does it mean to eat vegetables on a regular basis? Eat these foods in the same amount at the same frequency during the week.  4) Hello (patient's name)! Foods rich in vitamin K can alter the effect of Marevan®. Are you taking good care of your food?  5) Hello (patient's name)! Green leafy vegetables (spinach, ora-pro-nobis, lettuce, arugula, kale and taioba) are rich in vitamin K and can alter the effect of Marevan®.  6) Hello (patient's name)! Never stop eating green leafy vegetables, but always eat these foods in the same amount during the week.  7) Hello (patient's name)! If you are unable to eat green leafy vegetables daily, choose a weekly frequency and keep it that way. Avoid changes.  8) Hello (patient's name)! Eating the same regular amount of green leafy vegetables every week will help you control your INR.  9) Hello (patient's name)! Oils like soy, sunflower and olive oil are rich in vitamin K. Use as little as possible in food preparation. Avoid fried foods.  10) Hello (patient's name)! Leaf teas such as chamomile, matte or ginger tea can alter the control, and are not suitable for people using Marevan®.  11) Hello (patient's name)! Fruit teas (lemon, pineapple, apple) do not affect the control and can be taken normally.  12) Hello (patient's name)! Vitamins (tablets or capsules) are rich in vitamin K and can alter the effect of Marevan®. Avoid taking it without medical advice!  13) Hello (patient's name)! Oils and fats like soy, olive oil, mayonnaise and margarine have a lot of vitamin K. Use the least amount possible when cooking.  14) Hello (patient's name)! Be careful not to overdo it during the holidays, birthdays, or vacations. Remember to stick to your diet and don't drink alcoholic beverages.  15) Hello (patient's name)! Did you know that avocado, açaí and kiwi are rich in vitamin K? The day you eat any of these, avoid eating green leafy vegetables.  16) Hello (patient's name)! Inform your doctor if you are using a dietary supplement, vitamin, or herbal teas. These products can change the INR.  17) Hello (patient's name)! Whenever there are changes in your diet, remember to notify the health professional who accompanies the control of Marevan®.  18) Hello (patient's name)! Industrialized foods based on oil or herbs (salad dressing, packaged soup, tablet seasoning) can alter the INR.  19) Hello (patient's name)! Did you know that drinking alcohol with Marevan® greatly increases the risk of bleeding? Be careful and avoid drinking alcohol.  20) Hello (patient's name)! The effect of Marevan® may decrease when there is weight gain, diarrhea, or vomiting. See your doctor if there are any of these variations.  21) Hello (patient's name)! Take care with the use of sharp instruments (knives, scissors, or razors), as there is a risk of cutting with increased bleeding.  22) Hello (patient's name)! Physical activity is important but be careful regarding sports with risk of getting hit by other participants, such as football or volleyball.  23) Hello (patient's name)! Avoid daily activities with risk of cuts, falls or bumps. If there is bleeding, it can be more difficult to get it to stop.  24) Hello (patient's name)! Prefer low-impact physical activities (walking, water aerobics, lian gong), as there is less risk of hitting and bleeding. |
| **Closure:**  1) Hello (patient's name)! The messaging program ends today but keep on striving to be more and more successful in your treatment with Marevan®!  2) Hello (patient's name)! You will receive a call from the project team to get your opinion on these messages. Thank you for your cooperation! |

***Message tailored for the INR target of the patient. INR: international normalized ratio.

**Supplementary Box 2.**  Long-term interview

| 1. Did you receive text messages about your anticoagulation treatment and MarevanⓇ? What do you remember about the messages you received from our project last year? |
| --- |
| 2. Do you consider that something has changed in your treatment after you received the text messages? If yes, what has changed? |
| 3. For some reason you did not take MarevanⓇ (warfarin)? What are the reasons? |
| 4. Did you change your eating habits after the guidelines of our messages? What has changed? |
| 5. Did you start doing some physical activity after receiving the messages? If yes, which ones? If not, why? |
| 6. Do you remember the relationship between using MarevanⓇ and drinking alcoholic beverages? Did this information impact your alcohol consumption in any way? |
| 7. What would you say you learned about the treatment through the messages? |
| 8. Do you have WhatsApp? Would you like to receive our messages? (or, would you like to receive our messages again? |
| 9. How do you prefer to receive the intervention? Via audio or via text messages? |

**Supplementary Box 3.** Examples of translated COVID-19 SMS sent to patients

| 1) Hello (patient)! You will receive information about the new coronavirus infection (COVID-19). Stay tuned to the content!  2) Do you know what coronavirus is? It is a family of viruses that cause respiratory infections. The new coronavirus causes COVID-19 disease.  3) Hello [patient]! Coronavirus is transmitted by droplets that spread through the environment when the patient speaks, coughs or sneezes.  4) Hello [patient]! There are some people who may have a higher risk of getting severe coronavirus infection, so they are in “the risk group”.  5) Hello [patient]! As if you have a heart problem, be aware, because you may be at the risk group for coronavirus infection.  6) Hello [patient]! To prevent against coronavirus, avoid leaving the house and wash your hands often using soap and water or alcohol gel.  7) Hello [patient]! Wash your hands with soap and water, spreading all over the hand and fingers for at least 20 seconds.  8) Hello [patient]! Fever, cough, stuffy and runny nose, sore throat, feeling tired and shortness of breath may be symptoms of COVID-19.  9) Hello [name]! If you have flu-like symptoms with a fever that does not get better after 3 days, or run out of breath, seek medical attention immediately.  10) Hello [name]! If you have respiratory symptoms, wear a mask before leaving home. Remember to only go out if you require medical care.  11) Hello [patient]! Surgical masks effect's wear off after 2 hours of use or when wet. Use only once and then throw it away.  12) Hello [patient]! When using masks, remember to not touch its front. Put it on and take it off using the side bands.  13) Hello [patient]! Staying home is essential to avoid the coronavirus infection.  14) Hello [patient]! There still is no treatment for COVID-19. Drink water, rest and look for medical assistance if you have any warning signals.  15) Hello [patient]! When coughing or sneezing, cover your nose and mouth. This helps avoiding the virus' transmission to others.  16) Hello! If someone from you home has any symptoms, respect the quarantine period, avoid getting close to them and use different utensils, such as forks, spoons, and glasses.  17) Hello [patient]! It is important to clean the house and surfaces using water and soap, alcohol 70% or bleach with water.  18) Hello [patient]! Avoid touching your eyes, nose, and mouth with dirty hands or whenever you are not home.  19) Hello [patient]! Avoid close contact with people who have flu or cold-like symptoms, even if they live in your household.  20) Hello! Did you know that your mobile phone can be contaminated? Clean it regularly using 70% alcohol gel or diluted sanitary water.  21) Hello! Be careful with information you receive from WhatsApp and other social media, they are not always true! Always check if the source is reliable! |
| --- |

**Supplementary Box 4. Description of the HAS-BLED score**

| **Risk factors** | **Points** |
| --- | --- |
| H – Hypertension | 1 |
| A - Abnormal liver or renal function | 1 or 2 |
| S – Stroke | 1 |
| B – Bleeding | 1 |
| L – Labile INR | 1 |
| E – Elderly (< 65 years-old) | 1 |
| D – Drugs or alcohol | 1 or 2 |

0 – 2 points: low risk of bleeding, ≥ 3: high risk of bleeding

**Supplementary Box 5. Description of the CHA2DS2-VASc score**

| **Risk factors** | **Points** |
| --- | --- |
| Congestive heart failure | 1 |
| Hypertension | 2 |
| Age ≥ 75 years-old | 1 |
| Diabetes | 1 |
| Stroke | 2 |
| Vascular diseases | 1 |
| Age 65 – 74 | 1 |
| Female | 1 |

Stroke risk: 0 points: low, 1: moderate, ≥ 2 points: high risk

**Supplementary Box 6**. Examples from guidelines to final messages

| **Recommendation** | **Source** | **Final SMS** |
| --- | --- | --- |
| In patients with DVT treated with warfarin, the dose must be adjusted in view of reaching INR between 2.0 and 3.0 (target INR: 2.5) | 2013 Brazilian guidelines on platelet antiaggregants and anticoagulants in cardiology | Hello (patient's name)! When the INR is within the recommended range (2.0 to 3.0) the blood is neither "thick" nor too "thin", this is ideal! |
| Alcohol excess is a risk factor for bleeding in anticoagulated patients, mediated by poor adherence, liver disease, variceal bleeding, and risk of major trauma. Severe alcohol abuse and binge drinking habits should be corrected in patients eligible for OAC. | 2016 ACC/AHA Clinical Performance and Quality Measures for Adults With Atrial Fibrillation or Atrial Flutter | Hello (patient's name)! Did you know that drinking alcohol with Marevan® greatly increases the risk of bleeding? Be careful and avoid drinking alcohol. |
| Among patients treated with warfarin, the INR should be determined at least weekly during initiation of antithrombotic therapy and at least monthly when anticoagulation (INR in range) is stable. | 2016 ESC Guidelines for the management of atrial fibrillation developed in collaboration with EACTS | Hello (patient's name)! How often does the INR test need to be done? It depends on the case! Follow the guidance of the professional who controls it. |
